# Supplementary material for: Neurostructural associations with traumatic experiences during child- and adulthood
Source: Transl Psychiatry. 2022 Dec 15;12:515. doi: 10.1038/s41398-022-02262-9 (PMC9751132; doi:10.1038/s41398-022-02262-9)
Supplement: Supplementary file 1 — Suppl. Table 1 [file 41398_2022_2262_MOESM1_ESM.docx]

**Suppl. Table 1.** Detailed clinical characteristics of samples

**Comorbidities**

|  |  | **PTSD**_child_  **[n=25]** | | **PTSD**_adult_  **[n=26]** | |
| --- | --- | --- | --- | --- | --- |
|  |  | According to DSM-5 | | According to DSM-4 | |
|  |  | remitted | recurrent | remitted | recurrent |
| Mood disorders | Major depressive disorder | 7 | 14 | 2 | 11 |
|  | Bipolar disorder | 1 | 6 | 1 | - |
| Alcohol Use disorder | Alcohol abuse or dependency | 13 | 2 | - | 3 |
| Anxiety disorders | Panic disorder (w/wo Agoraphobia) | 4 | 3 | - | 3 |
|  | Agoraphobia (wo Panic disorder) | - | 2 | - | 1 |
|  | Social anxiety disorder | 11 | 10 | - | - |
|  | Specific phobia | 5 | 4 | - | 3 |
|  | Obsessive compulsive disorder | 4 | 4 | - | - |
|  | Generalized Anxiety Disorder | 1 | - | - | 3 |
| Somatization disorder | Somatization disorder | 1 | 1 | - | - |
| Eating disorder | Bulimia nervosa | 3 | 2 | 1 | 2 |
|  | Binge eating disorder | 3 | 3 | - | - |
|  |  |  |  |  |  |
| Personality disorders | Borderline Personality Disorder | 16 | |  |  |
|  | Other |  |  | 5 | |

**Medication**

|  | **PTSD**_child_  **[n=25]** | **PTSD**_adult_  **[n=26]** |
| --- | --- | --- |
| Antidepressant | 16 | 9 |
| Neuroleptics | 9 | 6 |
| Mood Stabilizer | - | - |
| Benzos | 2 | - |
| Other Psychotrop Med. | 4 | 6 |
| Other Non-Psychotrop Med. | - | 5 |

**Education**

|  | **PTSD**_child_  **[n=25]** | **PTSD**_adult_  **[n=26]** |
| --- | --- | --- |
| No graduation/ Still at school | 2 | 1 |
| Junior High School [Hauptschule] | 5 | 4 |
| Junior High School [Realschule] | 10 | 8 |
| A-Level/American SAT [Abitur] | 8 | 12 |
| Other |  |  |
