# Supplementary material for: Neurostructural associations with traumatic experiences during child- and adulthood
Source: Transl Psychiatry. 2022 Dec 15;12:515. doi: 10.1038/s41398-022-02262-9 (PMC9751132; doi:10.1038/s41398-022-02262-9)
Supplement: Supplementary file 2 — Suppl. Table 2 [file 41398_2022_2262_MOESM2_ESM.docx]

|  |  | **Groups** | | | | | | | | | | | |
| --- | --- | --- | --- | --- | --- | --- | --- | --- | --- | --- | --- | --- | --- |
| **Anatomical region** | **Hemisphere** | childhood | | | | | | adulthood | | | | | |
|  |  | PTSD_child_  [n=25] | | TC_child_  [n=26] | | HC_child_  [n=26] | | PTSD_adult_  [n=26] | | TC_adult_  [n=26] | | HC_adult_  [n=26] | |
|  |  | M | SD | M | SD | M | SD | M | SD | M | SD | M | SD |
| Amygdala | left | 0.91 | 0.07 | 0.95 | 0.07 | 0.98 | 0.10 | 1.02 | 0.10 | 1.02 | 0.10 | 0.93 | 0.08 |
|  | right | 0.87 | 0.07 | 0.92 | 0.08 | 0.93 | 0.11 | 1.00 | 0.11 | 0.98 | 0.10 | 0.92 | 0.08 |
| IFOG | left | 1.34 | 0.23 | 1.54 | 0.25 | 1.53 | 0.22 | 1.26 | 0.24 | 1.58 | 0.28 | 1.48 | 0.24 |
|  | right | 1.43 | 0.20 | 1.55 | 0.22 | 1.52 | 0.25 | 1.54 | 0.26 | 1.54 | 0.22 | 1.44 | 0.20 |
| Hippocampus | left | 3.06 | 0.28 | 3.14 | 0.29 | 3.23 | 0.27 | 3.24 | 0.27 | 3.24 | 0.31 | 3.09 | 0.24 |
|  | right | 3.36 | 0.31 | 3.46 | 0.32 | 3.51 | 0.32 | 3.54 | 0.28 | 3.54 | 0.36 | 3.37 | 0.26 |
| Ant. Insula | left | 4.59 | 0.42 | 4.89 | 0.48 | 4.81 | 0.59 | 4.70 | 0.53 | 5.05 | 0.67 | 4.60 | 0.45 |
|  | right | 4.61 | 0.47 | 4.86 | 0.45 | 4.85 | 0.59 | 4.71 | 0.50 | 4.91 | 0.64 | 4.55 | 0.42 |
| Pos. Insula | left | 2.28 | 0.19 | 2.36 | 0.23 | 2.33 | 0.28 | 2.22 | 0.24 | 2.35 | 0.33 | 2.15 | 0.25 |
|  | right | 2.53 | 0.23 | 2.62 | 0.32 | 2.60 | 0.31 | 2.57 | 0.30 | 2.72 | 0.38 | 2.51 | 0.30 |
| ACG | left | 5.28 | 0.51 | 5.45 | 0.60 | 5.74 | 0.76 | 5.47 | 0.59 | 5.72 | 0.77 | 5.43 | 0.62 |
|  | right | 4.01 | 0.52 | 4.03 | 0.43 | 4.24 | 0.68 | 3.87 | 0.55 | 4.11 | 0.69 | 3.84 | 0.52 |
| MTG | left | 13.42 | 1.42 | 13.69 | 1.61 | 14.12 | 1.50 | 14.49 | 1.51 | 14.85 | 1.42 | 14.07 | 1.46 |
|  | right | 13.67 | 1.56 | 13.85 | 1.65 | 14.37 | 1.52 | 14.54 | 1.44 | 14.71 | 1.51 | 13.93 | 1.40 |
| SFG | left | 13.91 | 1.27 | 14.50 | 1.95 | 14.65 | 1.71 | 14.23 | 1.43 | 14.75 | 1.86 | 13.84 | 1.68 |
|  | right | 13.69 | 1.32 | 14.49 | 1.93 | 14.33 | 1.72 | 13.99 | 1.28 | 14.51 | 1.88 | 13.93 | 1.40 |

**Suppl. Table 2.** Results of VBM analysis.

[**Abbreviations:** ACG – Anterior Cingulate Gyrus; Ant. Insula – Anterior insula; IFOG – Inferior fronto-orbital gyrus; MTG – Middle temporal gyrus; Pos. Insula – Posterior Insula; SFG – Superior frontal gyrus]
