# Supplementary material for: Neurostructural associations with traumatic experiences during child- and adulthood
Source: Transl Psychiatry. 2022 Dec 15;12:515. doi: 10.1038/s41398-022-02262-9 (PMC9751132; doi:10.1038/s41398-022-02262-9)
Supplement: Supplementary file 3 — Suppl. Table 3 [file 41398_2022_2262_MOESM3_ESM.docx]

|  |  | **Groups** | | | | | | | | | | | |
| --- | --- | --- | --- | --- | --- | --- | --- | --- | --- | --- | --- | --- | --- |
|  |  | childhood | | | | | | adulthood | | | | | |
|  |  | PTSD_child_  [n=25] | | TC_child_  [n=26] | | HC_child_  [n=26] | | PTSD_adult_  [n=26] | | TC_adult_  [n=26] | | HC_adult_  [n=26] | |
|  |  | M | SD | M | SD | M | SD | M | SD | M | SD | M | SD |
| TIV |  | 1443.00 | 103.09 | 1425.88 | 113.08 | 1473.95 | 113.62 | 1519.00 | 125.01 | 1508.02 | 138.11 | 1444.91 | 93.34 |

**Suppl. Table 3a.** Results of VBM analysis.

[**Abbreviations:** TIV – Total intracranial volume]

|  | **Analysis** | **Post-Hoc t-tests** | | | | | | | | | | |
| --- | --- | --- | --- | --- | --- | --- | --- | --- | --- | --- | --- | --- |
|  |  | **effect** | **Group** | **contrast** | **M_Diff_** | **CI [-95%; +95%]** | **T** | | **df** | **P_bon_cor_** | **Hedges‘g** |  |
| TIV | F_group_(2, 149) = 0.48, p=.62, η^2^<.01  **F_sample_(1, 149) = 5.34, p=.02,** **η^2^=.04**  **F_group x sample_(2, 149) = 3.81, p=.02,** **η^2^=.05** | sample |  | adulthood > childhood | 43.0 | 5.83; 80.1 | 2.29 | 307 | | .024 | 0.37 |  |

**Suppl. Table 3b.** Results of ANCOVA on TIV.

[**Abbreviations:** TIV – Total intracranial volume]
