# Supplementary material for: Neurostructural associations with traumatic experiences during child- and adulthood
Source: Transl Psychiatry. 2022 Dec 15;12:515. doi: 10.1038/s41398-022-02262-9 (PMC9751132; doi:10.1038/s41398-022-02262-9)
Supplement: Supplementary file 4 — Suppl. Table 4 [file 41398_2022_2262_MOESM4_ESM.docx]

|  | **pre-school** | **latency** | **prepubertal** | **pupertal** | **adolescent 1** | **adolescent 2** | **early adulthood** | **early adulthood 2** | **adulthood 1** | **adulthood 2** |
| --- | --- | --- | --- | --- | --- | --- | --- | --- | --- | --- |
| **Age (in yrs.)** | **0-5** | **6-8** | **9-10** | **11-13** | **14-16** | **17-21** | **22-25** | **26-30** | **31-35** | **>35** |
| **N** | 8 | 3 | 3 | 7 | 4 | 5 | 6 | 3 | 4 | 7 |

**Suppl. Table 4.** Age groups defined across both samples.
