# Supplementary material for: Neurostructural associations with traumatic experiences during child- and adulthood
Source: Transl Psychiatry. 2022 Dec 15;12:515. doi: 10.1038/s41398-022-02262-9 (PMC9751132; doi:10.1038/s41398-022-02262-9)

**Suppl. Figure 1.** Volumetric differences in the inferior fronto-orbital gyrus (IFOG), anterior cingulate gyrus (ACG), anterior (ant.) and posterior (pos.) insulae for both patient groups (PTSD_adult_, PTSD_child_) in time bins defined by the age of the index trauma separately for each hemispheres (left, right) in cm^3^.


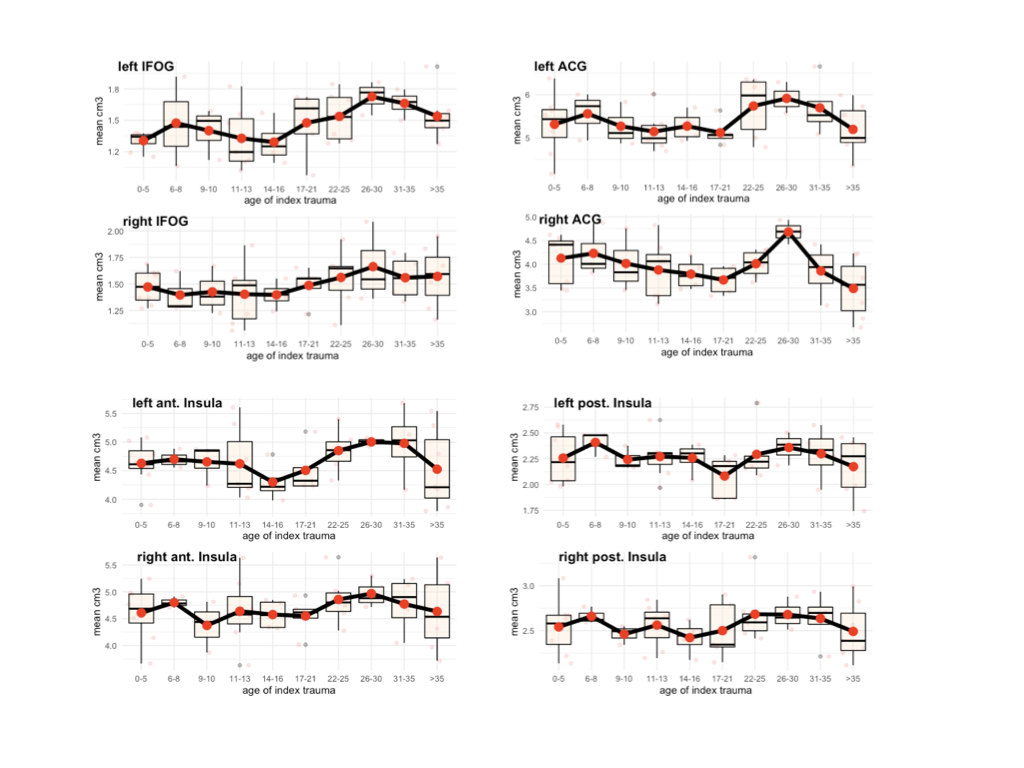

Supplement: Supplementary file 5 — Suppl. Figure 1 [file 41398_2022_2262_MOESM5_ESM.docx]
