# Supplementary material for: Neurostructural associations with traumatic experiences during child- and adulthood
Source: Transl Psychiatry. 2022 Dec 15;12:515. doi: 10.1038/s41398-022-02262-9 (PMC9751132; doi:10.1038/s41398-022-02262-9)

**Suppl. Figure 2.** Volumetric differences in the middle temporal gyrus (MTG) and superior frontal gyrus (SFG) for both patient groups (PTSD_adult_, PTSD_child_) in time bins defined by the age of the index trauma separately for each hemispheres (left, right) in cm^3^.


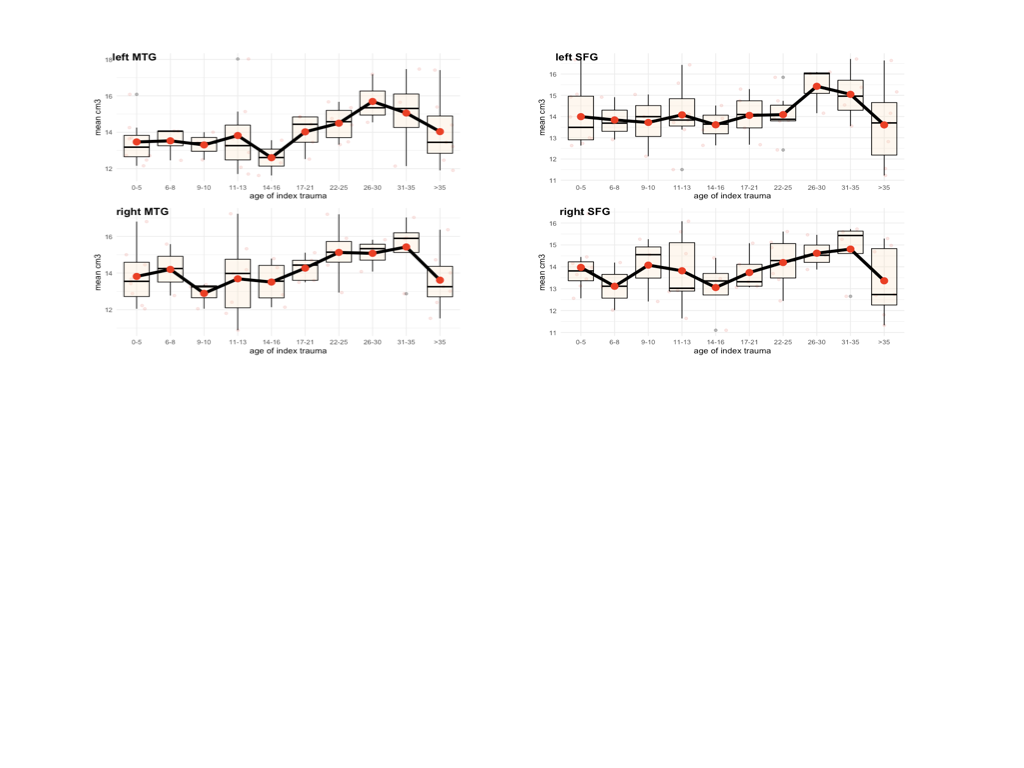

Supplement: Supplementary file 6 — Suppl. Figure 2 [file 41398_2022_2262_MOESM6_ESM.docx]
