# Supplementary material for: Neurostructural associations with traumatic experiences during child- and adulthood
Source: Transl Psychiatry. 2022 Dec 15;12:515. doi: 10.1038/s41398-022-02262-9 (PMC9751132; doi:10.1038/s41398-022-02262-9)

**Suppl. Figure 3.** Volumetric differences in total intracranial volume (TIV) between samples (childhood, adulthood), groups (PTSD, TC, HC) and hemispheres (left, right) in cm^3^.


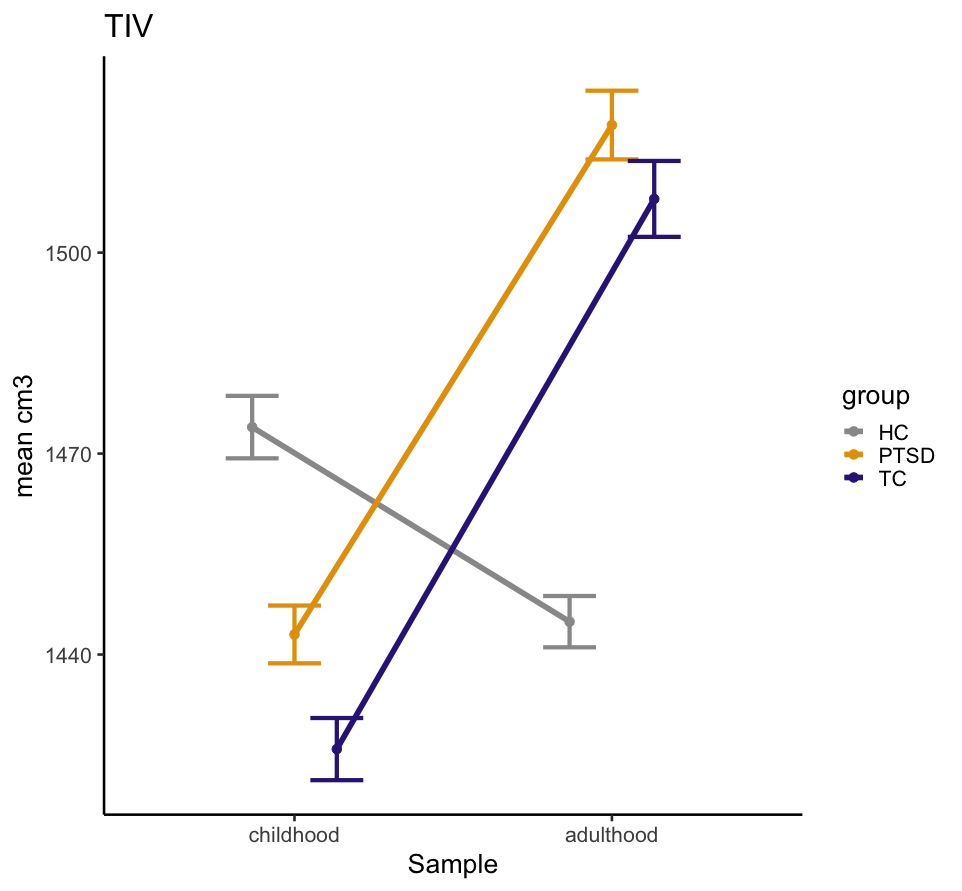

Supplement: Supplementary file 7 — Suppl. Figure 3 [file 41398_2022_2262_MOESM7_ESM.docx]
